# The current status of old traditional medicine introduced from Persia to China

**DOI:** 10.3389/fphar.2022.953352

**Published:** 2022-09-14

**Authors:** Jinmin Shi, Yifan Yang, Xinxin Zhou, Lijun Zhao, Xiaohua Li, Abdullah Yusuf, Mohaddeseh S. M. Z. Hosseini, Fatemeh Sefidkon, Xuebo Hu

**Affiliations:** ^1^ College of Plant Science and Technology, Innovation Academy of International Traditional Chinese Medicinal Materials, National-Regional Joint Engineering Research Center in Hubei for Medicinal Plant Breeding and Cultivation, Medicinal Plant Engineering Research Center of Hubei Province, Institute for Medicinal Plants, Huazhong Agricultural University, Wuhan, China; ^2^ Department of Pharmacy, Renmin Hospital, Hubei University of Medicine, Shiyan, China; ^3^ College of Chemistry and Environmental Science, Laboratory of Xinjiang Native Medicinal and Edible Plant Resources Chemistry. Kashi University, Kashgar, China; ^4^ Research Institute of Forests and Rangelands, Tehran, Iran

**Keywords:** Compendium of Materia Medica, Persian, Iran, Chinese herbal medicine, the Silk Road, traditional Chinese medicine

## Abstract

Traditional Chinese medicine (TCM) includes over ten thousand herbal medicines, some of which were introduced from outside countries and territories. The Silk Road enabled the exchange of merchandise such as teas, silks, carpets, and medicines between the East and West of the Eurasia continent. During this time, the ‘Compendium of Materia Medica’ (CMM) was composed by a traditional medicine practitioner, Shizhen Li (1,518–1,593) of the Ming Dynasty. This epoch-making masterpiece collected knowledge of traditional medical materials and treatments in China from the 16th century and before in utmost detail, including the origin where a material was obtained. Of 1892 medical materials from the CMM, 46 came from Persia (now Iran). In this study, the basic information of these 46 materials, including the time of introduction, the medicinal value in TCM theory, together with the current status of these medicines in China and Iran, are summarized. It is found that 20 herbs and four stones out of the 46 materials are registered as medicinal materials in the latest China Pharmacopoeia. Now most of these herbs and stones are distributed in China or replacements are available but saffron, ferula, myrrh, and olibanum are still highly dependent on imports. This study may contribute to the further development, exchange, and internationalization of traditional medicine of various backgrounds in the world, given the barriers of transportation and language are largely eased in nowadays.

## 1 Introduction

**FIGURE 1:**
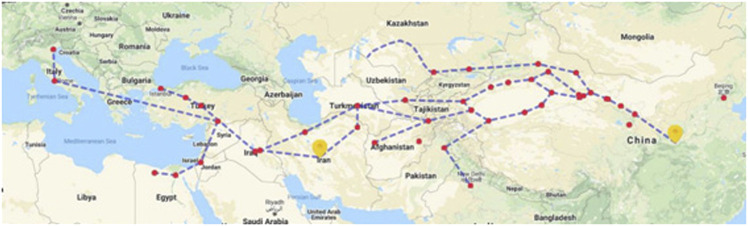
Illustrative map of the Silk Road.

Persia (present Iran) was an important territory along the ancient Silk Road. The Silk Road was first officially proposed by a German geographer Ferdinand von Richthofen in 1877 (Wu, 2019). The Silk Road started from Chang’an (now Xi’an city of China) in the Western Han Dynasty and crossed the Longshan Mountains and Hexi Corridor, and reached to Xinjiang through the Yumenguan and Yangguan passes. Then, passing through modern-day Tajikistan, Turkmenistan, Iran, Iraq, Turkey, it finally reached to Africa and Europe. The Silk Road is a historical route that traverses Eurasia and promotes friendly exchanges between Europe and Asia (Figure 1). In the trade along the Silk Road, China’s export of silk is the most representative; hence, it was named “Silk Road.” Businessmen and messengers from all over the world have continued to carry out economic and cultural exchanges along this road.

The record of Iran in China can be dated back to before Christ (BC), corresponding to the Arsa Dynasty of the Parthian Empire (247 BC–226 AD) in Iran (Lei, 2011). In 138 BC, Qian Zhang was sent as an envoy to greet the Western territories. Qian Zhang opened up a new era of goods and culture exchanges between old China and foreign countries and territories. The route was historically visualized as the “Qian Zhang short-cut.” With the gradual prosperity of the Silk Road, China’s relations with Persia became increasingly close, and many medicinal materials from Persia have also been introduced to China.

The Compendium of Materia Medica (CMM; “本草纲目” in Chinese characters or “Bencao Gangmu” in the Chinese phonetic alphabet) provides a body of medicinal products and agents that was compiled by Shizhen Li (1,518–1,593) in the Ming Dynasty. He devoted his entire life and spent 30 years to complete the masterpiece. The book is divided into 16 sections (waters, fires, earth, metals, stones and minerals, herbs, cereals, vegetables, fruits, wood, fabrics and utensils, insects, worms and amphibians, animals with scales, shells, fowls, animals and humans). With a total of 1.9 million Chinese characters, the book collected 1892 medicines, 11,096 medical prescriptions, and 1,160 illustrations. The book systematically describes the knowledge of each CMM medicine in detail and provides an explanation of the names and background of each medicinal material and it discusses quality, taste, indications, explication, prescriptions, etc. The number of medicinal herbs recorded and the details of the content in the CMM are vast and during the preparation of CMM, Shizhen Li visited a wide range of regions and consulted many people in order to ensure authenticity and accuracy in the recording of ethnobotanical information. He summarized and criticized herbal books written by previous scholars, corrected errors in these books, and then invented a comparative system of drug classification methods. In classifying medicinal materials, he established a system from low level to advanced, from simple to complex, which was a very advanced classification method at that time. The first edition CCM was published over 400 years ago. Since then, it has been translated into other languages, making it accessible to the rest of the world. As a world’s documentary heritage, the CMM was successfully selected in the Memory of the World Register in 2011 (Zhang and Zheng, 2016). Being a treasure of Chinese medicine for thousands of years in China, the CMM is broad and profound. Its scientific achievements encompass many aspects of ancient science and technology but it still has great relevance in modern times as it covers botany, geography, meteorology, mineralogy, biology, physics, etc. Although CMM is primarily a medical book, it is worth studying in many subject fields. The CMM has a far-reaching impact on the development of Chinese medicine and other disciplines.

It has a long history of cultivating medicinal plants in China. These plants account for a major part of traditional Chinese medicine (TCM), however, not all of these herbs are native to China. As exotic plants, they were incorporated and developed into the TCM system despite being introduced to China from overseas. These exotic plants have become naturalized, gradually adapting to local environments and are now widely recognized by Chinese people. According to Tan and Peng (2014), the CMM contained 96 foreign medicines imported from different countries, of which, 46 were imported from Persia. It accounts for nearly half of the number of foreign drugs, including nine herbs, 12 types of wood, seven types of fruit, 15 metals and minerals, two types of vegetables, and one fowl. The exchange of medicinal materials along the ancient Silk Road is a great contribution to the development of traditional Chinese medicine, moreover, it also links the civilizations between Chinese and abroad. In this review study, with these 46 Persian imported medicines as a focus, we endeavored to trace the nomenclature, distribution, pharmacology, and corresponding disease treatments, and most importantly, the changes and evolution of these characteristics in 2 thousand years for each of the materials. The historical, medical, economic, and cultural significance, wherever necessary, were also discussed.

## 2 Materials and methods

All original information on the medicinal materials was based on a Chinese version of the Compendium of Materia Medica (Li, 2005), of which an English version of the book is also available (Li, 2003). Any TCM documented in the CMM with an origin of Chinese character “波斯” (“Bosi” in Chinese phonics) was thought to be imported from Persia. All TCMs from the CMM were compared with the 2020 edition of the Pharmacopoeia of People’s Republic of China (PPRC) [Chinese version] (Commission, 2020) or 2015 edition of the Pharmacopoeia of People’s Republic of China (ePPRC)[English version] (Commission, 2017). More literature on pharmacology, history, and botany of the plants, were obtained by searching against the following databases: Scifinder, PubMed, and Google Scholar. The Royal Botanic Gardens, Kew (www.mpns.science.kew.org) was used to check the correctness of the nomenclature of the reported plant species.

## 3 Results

A total of 46 TCMs documented in the CMM were clearly mentioned to have an origin linked to Persia. The name and current distribution of these TCMs are included in Table 1. The CMM was published 500 years ago, but most of the TCMs noted in the book were introduced to China much earlier. In a search of the possible origin, 11 TCMs were introduced to China before Qin and Han dynasties (before 220 AD), 12 were introduced to China in the Wei, Jin, and Nanbei dynasties (220–589 AD), 20 are associated with Sui, Tang, and Five dynasties (618–907 AD). Only three were introduced from the Song to Ming dynasties (960–1644 AD), indicating that the TCM commerce in the Silk Road was established over a thousand of years (Table 2).

**TABLE 1:** Medicinal plants exported from Persia to old China as recorded in the Compendium of Materia Medica. The current distribution of these plants is noted.

Medicine name	Current distribution^a^			Reference
	China	Iran	Rest of the world	
Agarwood-*Aquilaria malaccensis* Lam	ND	ND	Bangladesh, Bhutan, India, Indonesia, Iran, Malaysia, Myanmar, Philippines, Singapore, and Thailand	Naziz et al. (2019)
Aloe-*Aloe ferox* Mill	CT	ND	Africa	He et al., 2016; Wintola and Afolayan, 2014
Aloe-*Aloe vera* (L.) Burm.f	CT	WD, CT	India, Malaysia, Africa, Mexico, Saudi Arabia, United States, etc.	He et al., 2016; Avijgan et al., 2016; Dey et al., 2022; Bieski et al., 2015
Babchi*-Cullen corylifolium* (L.) Medik	WD, CT	ND	India, Burma, Vietnam, Sri Lanka	Ren et al., 2020; Sharifi-Rad et al., 2020
Benzoin-*Styrax tonkinensis* (Pierre) Craib ex Hartwich	CT	ND	Vietnam, Thailand, etc.	Sun et al. (2022)
Betel pepper-*Piper betle* L	WD, CT	ND	India, Sri Lanka, Vietnam, Malaysia, Indonesia, the Philippines, Burma, etc.	Yin et al., 2009; Sarkar et al., 2000
Black myrobalan-*Terminalia chebula* Retz	WD, CT	ND	India and Myanmar	Wu et al. (2020a)
Borneol-*Cinnamomum camphora* (L.) J. Presl	WD, CT	WD, CT	Bolivia, China, El Salvador, Gabon, Sri Lanka, Taiwan	Bieski et al. (2015)
*Carpesium abrotanoides* L	WD, CT	WD	Korea, China, Vietnam, Japan, India, Myanmar, and Russia	Ibrahim et al. (2022)
Chinese olive-*Canarium album* (Lour.) DC.	WD, CT	ND	Taiwan	Li et al., 2022a; Yeh et al., 2017
Dill-*Anethum graveolens* L	CT	WD, CT	India, Pakistan, United States, Mexico, Germany, and the Netherlands,	Tian, 2012; Sadeghi et al., 2021
Ebony-*Diospyros ebenum* J.Koenig ex Retz	WD	WD	Sri Lanka	Perera et al. (2018)
Ferula-*Ferula fukanensis* K.M. Shen	WD, CT	ND	ND	Ye et al. (2011)
Ferula-*Ferula sinkiangensis* K.M. Shen	WD, CT	ND	ND	Fan et al. (2021)
Fig-*Ficus carica* L	CT	WD, CT	Mediterranean coast, from Turkey to Afghanistan	Zou et al., 2020; Buso et al., 2020; Barolo et al., 2014
Figwort flower picrorhiza- *Neopicrorhiza scrophulariiflora* (Pennell) D.Y.Hong	WD, CT	ND	Nepal, north east India, Bhutan, northern Myanmar	Guo et al., 2020; Rokaya et al., 2020
Frankincense-*Boswellia sacra* Flück	ND	ND	Ethiopia, Somalia, Arabian Peninsula	Cao et al. (2019)
Golden shower-Cassia fistula L	CT	WD, CT	Burma, Sri Lanka, India	Tan et al., 2018; Antonisamy et al., 2019; Mwangi et al., 2021
Grape-*Vitis vinifera* L	CT	CT	Around the world	Meng et al., 2021); Pasrsa, 1960); Seo et al., 2021
Jackfruit-*Artocarpus heterophyllus* Lam	CT	ND	Tropical regions	Zhu et al., 2021; Gupta et al., 2022
Jasmine-*Jasminum sambac* (L.) Aiton	WD, CT	WD, CT	India and tropical regions of both hemispheres	Wan, 2014; Ghasemi Ghehsareh et al., 2015; Sengar et al., 2015
Large fruited elm-*Ulmus macrocarpa* Hance	WD	ND	Asia	Rho et al., 2019; Oh et al., 2008
Long pepper-*Piper longum* L	WD	ND	Tropical and subtropical regions of the world	Li et al. (2022b)
Marking nut tree-*Semecarpus anacardium* L.f	ND	ND	India, Ceylon, Burma	Pasrsa, (1960)
Myrrh-*Commiphora myrrha* (T.Nees) Engl	ND	ND	Somalia, Ethiopia, Arabian Peninsula, India	Parsa, 1959b; Latha et al., 2021
Natural indigo-*Isatis tinctoria* subsp*. tinctoria*	WD, CT	CT	ND	Wong et al., 2022; Pasrsa, 1960
Natural indigo-*Persicaria tinctoria* (Aiton) Spach [Polygonaceae]	WD, CT	ND	ND	Sun et al. (2021)
Natural indigo-*Strobilanthes cusia* (Nees) Kuntze	WD, CT	ND	India, Myanmar, Thailand	Chen et al. (2018b)
Nutgalls-*Quercus infectoria* G. Olivier	ND	WD	Greece, Asia Minor, Syria, etc.	Zin et al. (2019)
Phoenix date-*Phoenix dactylifera* L	CT	WD, CT	South America, Australia, South Asian, the Middle East, North Africa	Mao et al., 2019; Ataei et al., 2020; Gad El-Hak et al., 2022
Pistachio-*Pistacia vera* L	CT	WD, CT	Syria, Iraq, America, Afghanistan, Australia, Turkey, etc.	Fu et al., 2022; Pasrsa, 1960; Bailey and Stein, 2020
Saffron-*Crocus sativus* L	CT	WD, CT	Spain, Greece, Italy, and Nepal	Hu et al. (2020)
Spinach-*Spinacia oleracea* L	CT	CT	Around the world	Roberts and Moreau, (2016)
Villous amomum-*Wurfbainia villosa* (Lour.) Skornick. and A.D. Poulsen	WD, CT	ND	ND	Chen et al. (2020a)
Villous amomum-*Wurfbainia villosa* var. *xanthioides* (Baker) T.L.Wu and S.J.Chen	WD, CT	ND	Laos, Vietnam, Kampuchea, Thailand, India	Chen et al., 2020b; Li et al., 2018

a WD, wild growing; CT, cultivation; ND, no distribution.

**TABLE 2:** Chinese medicinal materials introduced from Persia to China in the Compendium of Materia Medica.

Time	Dynasty in China	Name of medicinal materials	References
Before 220 AD	Qin, Han, and before	Big fruit elm, cinnabar, alum stone, grape, fibroferrite, ostrich, aloe, chebule, spinach, coral, *Carpesium abrotanoides*	Zhao and Zhang, 2015; He and Zhang, 2018; Zhuang and Ling, 1980; Li and Zhu, 2005; Xiao and Yu, 1986; Chen and Chen, 1991
220–589 AD	Wei, Jin, and Nanbei	Betel, atacamite, nutgalls, frankincense, myrrh, sulfur, jasmine, salt, long pepper, ebony, Chinese eaglewood, *Psoralea corylifolia*	Jin and Guo, 2011; Li and Zhu, 2005; Wan, 2014; Cao, 2009
618–907AD	Sui, Tang, and Five dynasties	Benzoin, *Ferula asafoetida*, borneol, olive, dill, iron, figwort flower picrorhiza rhizome, phoenix date, silver, villous amomum fruit, gold, baladur, natural indigo, fig, pistachio, litharge, *Cassia fistula*, jackfruit, lead, copper	Zhuang and Ling, 1980; Li and Zhu, 2005; Huang and Xian, 1990; Teng, 2007
960–1279AD	Song	Silver ore	
1271–1368AD	Yuan	Saffron	Zhuang and Ling, (1980)
1368–1644AD	Ming	Calamine	

A detail of the basic information of the TCM with a registry of China Pharmacopoeia, is organized in Table 3. Because of the tradition of TCM, one material might be included in two registries with different materials, such as *Strobilanthes cusia* (Nees) Kuntze and this is used as a single medicine or in combination with *Persicaria tinctoria* (Aiton) Spach and *Isatis tinctoria* subsp*. tinctoria*. On the other hand, two different parts of one herb might be separately registered in the PPRC. The leaf and root of *Isatis tinctoria* subsp*. tinctoria* are separately registered in the PPRC as two medicines and their formulations are different. It is worth noting that one stone is not grouped in a formula but most of these materials are still in use with as many as 75 formulae being utilized. According to the CMM method, the TCM from Persia was divided into categories of herbs, vegetables, fruits, woods, metal/stone/mineral, and fowls. The following sections provide details in terms of these medicinal products.

**TABLE 3:** Basic information in China Pharmacopoeia on medicinal materials recorded in the Compendium of Materia Medica.

Common name	Scientific name	Medicinal part	Treatment^a^	Formula entry number^b^	Reference
Aloe ‘Luhui’	*Aloe vera* (L.) Burm.f. or *Aloe ferox* Mill	Leaf	Used for constipation due to heat binding, fright epilepsy and convulsion, chronic malnutrition in children. External use for tinea and sore	6	Bieski et al. (2015)
Alum stone-Alumen ‘Baifan’	*—*	—	External use for detoxifying and killing worms and relieving itching. Internal use for hemostasis and relieving diarrhea, dispelling anemogenous phlegm. External treatment for eczema, scabies, archoptoma, hemorrhoids, and suppurative otitis media. Oral administration for chronic diarrhea, hematochezia, metrorrhagia and metrostaxis, epilepsy	5	Hong et al. (2011)
Alum stone-Melanteritum ‘Lvfan’ or ‘zaofan’	*—*	—	Used for abdominal distension due to hookworm diseases, malnutritional stagnation and chronic dysentery, hematochezia with syndrome of intestinal wind, chlorosis due to blood deficiency, eczema and scabies, throat obstruction, and aphtha	2	Liu and Kong, (2007)
Baphicacanthus root ‘Nan Banlangen’	*Strobilanthes cusia* (Nees) Kuntze	Rhizome and root	Epidemic diseases, fever with sore throat, macula, and papule	1	Yu et al. (2021)
Benzoin	*Styrax tonkinensis* (Pierre) Craib ex Hartwich	Resin	Used for stroke and phlegm syncope, qi depression leading to cold extremities, apoplectic disease and coma, heart and abdominal pain, postpartum hemorrhagic syncope, acute infantile convulsion	9	Wong et al. (2022)
Calamine ‘‘Luganshi	*—*	—	Used for red eye and swollen sore, blepharitis marginalis, white membrane in eye, pterygium growing through the cornea, refractory ulcer, pus dripping wet, eczema pruritus	0	Meng et al. (2017)
Chinese eaglewood ‘Chenxiang’	*Aquilaria sinensis* (Lour.) Spreng	Trunk	Used for distention, oppression and pain of chest and abdomen, vomit and hiccup due to stomach cold, kidney deficiency, qi reversal and rapid panting	44	Yang et al. (2019)
Chinese olive ‘Qingguo’	*Canarium album* (Lour.) DC.	Fruit and oil from fruit	Used for swelling and pain in throat, cough with sticky sputum, vexation heat and thirst, allergy after eating fish and crab	6	Li et al., 2022a; Yeh et al., 2017
Cinnabar ‘Zhusha’	*—*	—	Used for palpitation and susceptibility to fright, insomnia and dreamful sleep, epilepsy and spasm, febrile convulsion, blurred vision, mouth sore, throat impediment, sore, and swelling sepsis	71	Wu et al. (2020b)
Common carpesium fruit ‘Heshi’	*Carpesium abrotanoides* L	Fruit	Used for ascariasis, enterobiasis, cestodiasis, and abdominal pain. Also for chronic malnutrition in children	1	Liu et al., 2010; Pu and Pu, 2012
Dyers wood leaf ‘Daqingye’	*Isatis tinctoria* subsp*. tinctoria*	Leaf	Warm disease symptoms: fever, coma, macule, eruption, mumps	27	Wong et al. (2022)
*Ferula asafoetida* ‘Awei’	*Ferula sinkiangensis* K.M. Shen or *Ferula fukanensis* K. M. Shen	Resin	Used for food retention, blood stasis, lump in abdomen, abdominal pain due to worm accumulation	5	Mala et al. (2018)
Figwort flower picrorhiza rhizome ‘Huhuanglian’	*Neopicrorhiza scrophulariiflora* (Pennell) D.Y.Hong	Rhizome	Used for hectic fever and tidal fever, infantile fever due to chronic, jaundice and reddish urine, hemorrhoid swelling	8	Jin et al. (2019)
Isatis root ‘Banlangen’	*Isatis tinctoria* subsp*. tinctoria*	Root	Pestilence, fever with sore throat, papule, and mumps	60	Wong et al. (2022)
Long pepper ‘Biba’	*Piper longum* L	Ear	Used for cold pain of epigastric, vomit, diarrhea, qi stagnation due to cold congealing, chest impediment and heart pain, headache, and toothache	12	Li et al. (2022b)
Malaytea scurfpea fruit ‘Buguzhi’	*Cullen corylifolium* (L.) Medik	Fruit	Used for insufficiency of kidney Yang, impotence and spermatorrhea, enuresis, frequent urination pain in waist and knees, dyspnea, early morning diarrhea. External use for white patch wind, alopecia areata	45	Sharifi-Rad et al., 2020; Khushboo et al., 2010; Ren et al., 2020
Medicinal terminalis fruit ‘Hezi’	*Terminalia chebula* Retz	Fruit	Used for chronic diarrhea and dysentery, bloody stool and rectal prolapse, lung deficiency, panting and coughing, chronic cough, angina and hoarseness	27	Sotoudeh et al. (2019)
Myrrh ‘Moyao’	*Commiphora myrrha* (T.Nees) Engl	Resin	Used for chest impediment and heart pain, stomach pain, dysmenorrhea and amenorrhea, postpartum stasis, painful abdominal mass, impediment pain, injury due to knocks and falls, carbuncle and swelling, sore	75	Lebda et al., 2021; Parsa, 1959b
Natural borneol ‘Tianran Bingpian’	*Cinnamomum camphora* (L.) J.Presl	Branch and leaf	Used for unconsciousness due to sexogenous febrile disease, eclampsia, stroke and phlegm syncope, qi depression leading to cold extremities, coma due to noxious pathogen attack, chest impediment and heart pain, red eye, mouth sore, swollen sore throat, purulent discharge in ear	4	Liu et al., 2021; Xiao et al., 2021; Zhang et al., 2021
Natural indigo ‘Qingdai’	*Strobilanthes cusia* (Nees) Kuntze, *Persicaria tinctoria* (Aiton) Spach or *Isatis tinctoria* subsp*. tinctoria*	Leaf or stem	Macula and papule caused by warm toxin, blood heat with hematemesis, chest pain with hemoptysis, mouth sore, mumps, sore throat	21	Wong et al., 2022; Sun et al., 2021
Olibanum ‘Ruxiang’	*Boswellia sacra* Flück	Resin	Chest qi disorder, heart pain, stomach pain, abdominal pain, traumatic injuries, sore, ulcer	89	Huang et al. (2022)
Saffron ‘Xihonghua’	*Crocus sativus* L	Stigma	Used for amenorrhea and pelvic mass, postpartum stasis, warm toxin and resolving macula, depression and lump with oppression, fright palpitation	7	Parsa, (1959b)
Western fruit ‘Xiqingguo’	*Terminalia chebula* Retz	Fruitlet	Yin deficiency diphtheria	7	Sotoudeh et al. (2019)
Villous amomum fruit ‘sharen’	*Wurfbainia villosa* (Lour.) Skornick and A.D. Poulsen	Fruit	Used for excessive water, gastric lump and no sense of hunger, deficient cold of spleen and stomach, vomit and diarrhea, morning sickness in pregnancy, restless fetus	66	Lu et al., 2018; Yue et al., 2021

a Based on China Pharmacopoeia (2020).

b The number of each material being used in TCM, formula of China Pharmacopoeia (2020).

### 3.1 The category of herbs

**Natural indigo**/*Strobilanthes cusia* (Nees) Kuntze, *Persicaria tinctoria* (Aiton) Spach, or *Isatis tinctoria* subsp*. tinctoria/*“Qing Dai,”“青黛”^1^

Natural indigo is used as a dry powder extracted from the leaves or stems of *Strobilanthes cusia* (Nees) Kuntze, *Persicaria tinctoria* (Aiton) Spach, or *Isatis tinctoria* subsp*. tinctoria.* Natural indigo was commonly used as a dye for cloth and paint in old times but it has been adopted as an important TCM for a long time. It is now included in the 2020 edition of the Pharmacopoeia of People’s Republic of China (PPRC), which is used for reducing body heat and detoxification of the body. *I. tinctoria* subsp*. tinctoria* produces a large variety of chemicals including terpenoids, alkaloids, organic acids, and others, which are the chemical foundation of multiple pharmacological effects including anti-inflammation, anti-tumor, anti-allergy, and anti-microbes (Wong et al., 2022). *I. tinctoria* subsp*. tinctoria* became a popular TCM in recent years to treat viruses (Mandal et al., 2021). The phenols, polysaccharides, lignans, indole alkaloids, and glycosidic bisindole alkaloids from the plant, were reported to exhibit efficacy against SARS coronavirus (Lin et al., 2005), hepatitis B virus (Wang et al., 2020), Japanese encephalitis virus (Chang et al., 2012), avian influenza A/B virus (Yang et al., 2013), and influenza virus A (Liu et al., 2015), respectively. *S. cusia* and *P. tinctoria* are not as commonly used in comparison to *I. tinctoria* subsp. *tinctoria* in TCM even though they are regarded as being important in PPRC. There are fewer studies that report on the pharmacological properties of these species. For example, a peptide derivative, aurantiamide acetate from *S. cusia* was shown to be anti-inflammatory and anti-viral (Zhou et al., 2017). The *I. tinctoria* subsp*. tinctoria* was also shown with a strong activity against the protease of SARS-CoV-2, the culprit of the ongoing COVID-19 pandemic (Mandal et al., 2021). A famous, ready-to-use Chinese formula *Lianghuaqingwen* capsule, which consists of the *I. tinctoria*, was in a clinical trial against COVID-19 (Lee et al., 2021). In addition to activity against viruses, *P. tinctoria* was shown to be effective against bacteria (Kataoka et al., 2001) and this was tested against *Helicobacter pylori*–infected Mongolian gerbils. This species also has been indicated to hold anti-melanogenesis (Chung et al., 2018) and anti-neuroinflammation actions (Lee et al., 2018).

Interestingly, the three medicinal plants are now all cultivated in China and *I. tinctoria* subsp*. tinctoria* has a wide application in TCM. *I. tinctoria* subsp*. tinctoria* is currently distributed in Iran; however, there are no reports of the distribution of *S. cusia* and *P. tinctoria* in Iran. Prepared in coarse or fine powder, the leaves of *I. tinctoria* subsp*. tinctoria* are sold as henna in Iran, which is mainly used as a cosmetic for coloring the skin (Pasrsa, 1960).

**Long pepper**/*Piper longum* L.*/*“Biba,”“荜茇”

Long pepper is a climbing vine tree that is known to be distributed in southern China but has not been recorded in Iran. In TCM, long pepper is the dried, nearly mature or mature ears of the *Piper longum* L. (Piperaceae). Shizhen Li in the CMM said that a soup made from boiling long pepper together with milk was excellent in treating headaches, rhinorrhea, and toothache. Long pepper is now included in the PPRC, with functions of warming the interior body, dissipating cold, and for stopping pain. Modern studies have shown that the main components of long pepper are amide alkaloids and volatile oils that have caryophyllene and pentadecane (both about 17.8%) and bisaboline (11%), as well as amino acids and inorganic elements (Li, 2017). Pharmacological studies have shown that long pepper has anti-bacterial and anti-viral properties. Also, it has the capacity for improving lipid and glucose metabolism while showing anti-inflammatory, anti-tumor, anti-gastric ulcer, anti-diarrhea, and anti-oxidant effects (Li D. et al., 2022).

**Figwort flower picrorhiza rhizome**/*Neopicrorhiza scrophulariiflora* (Pennell) D. Y. Hong/“Hu Huanglian,” “胡黄连”

The medicinal figwort flower is the dry rhizome of *Neopicrorhiza scrophulariiflora* (Pennell) D.Y. Hong, a plant of the Scrophulariaceae. It is now predominantly distributed in Tibet and is thus an important Tibetan medicine. There are no records of the plant being grown in Iran. It was listed as a national tertiary protection plant and recorded in the PPRC. In TCM theory, *N. scrophulariiflora* is used as a tonic for the liver and gallbladder and is said to improve the eyesight. It can also be utilized for treating fevers and it has a reputation as being used for diabetes. Apart from this, it may be used for treating convulsions in pregnant women. It stops diarrhea and dysentery and treats five types of hemorrhoids. Studies showed that its main chemical components are iridoids, cucurbitol, phenylethanol glycosides, and phenol glycosides (Guo et al., 2020). The pharmacological effects of figwort flower include lowering fat of the liver and gallbladder, antibacterial and anti-inflammatory effects, protection of damaged nerve cells, and apoptosis of cardiomyocytes (Jin et al., 2019).

**Villous amomum fruit**/*Wurfbainia villosa* (Lour.) Skornick. and A.D. Poulsen or *Wurfbainia villosa* var. *xanthioides* (Baker) T.L. Wu and S.J. Chen/“Sushami,”“缩砂蔤”

Villous amomum fruit is the dried, mature fruit of *W. villosa* or *W. villosa* var. *xanthioides*. It is included in the PPRC with the name of Sharen*.* Both wild and cultivated *W. villosa* are distributed in China but there are currently no records that indicate it being grown in Iran. The main components of *W. villosa* are volatile essential oils, such as borneol acetate, camphor, borneol, β-bisabolene, nerolidol, and cubeno (Chen et al., 2020a). There are also nonvolatile components including polysaccharides, flavonoid glycosides, organic acids, and inorganic components (Li et al., 2018). It has been disclosed that *W. villosa* has various pharmacological effects including gastrointestinal protection of anti-ulcer effects, promoting gastric emptying and gastric peristalsis (Lu et al., 2018; Yue et al., 2021). Other studies have shown this species possesses functions of anti-inflammation (Yin et al., 2019), anti-diarrhea, and anti-obesity (Kim et al., 2022), as well as bacteriostatic effects (Tang et al., 2021). *W. villosa* has also been extensively utilized for a range of bowel diseases (Chen et al., 2018a).

**Betel**/*Piper betle* L./“Jujiang,”“蒟酱”

Betel is a plant that has a long history as a TCM but it is not included in PPRC. Shizhen Li confirmed that Jujiang was made from *Piper betle* L. but not Biba (*Piperis longum* L.). Many studies have indicated that betel has a potential for treatment of *Leishmania donovani*– induced parasitic disease (Misra et al., 2009). It has also been indicated with a profile of anti-fertility (Sarkar et al., 2000). Other medicinal uses include its importance as an anti-malarial(Tamura et al., 2020), anti-gout (Vikrama Chakravarthi et al., 2022), anti-oxidant (Vikrama Chakravarthi et al., 2022), anti-platelet, and anti-inflammation remedy (Saeed et al., 1993). Yin et al. (2009) isolated and identified amide alkaloids, triterpenoids, lignin, flavonoids, and sterols from the stems of the plant. Appreciated for the function of improving the memory, the betel, which was imported from India, is sold under the name of Tanbool or Tanflool in Iran (Pasrsa, 1960).

**Babchi*/*** *Cullen corylifolium* (L.) Medik./“Buguzhi,” “补骨脂”

Babchi is produced from the dry ripening fruits of *Cullen corylifolium* (L.) Medik plants. The fruit is harvested and dried after it matures in autumn, and the fruit is used as a medicine. It is included in PPRC for treating skin diseases such as psoriasis. *C. corylifolia* also has a long history in Ayurvedic medicine. It is widely distributed in India. Babchi contains various chemical components including coumarins, monoterpenoids, flavonoids, and benzofurans, and has a wide range of pharmacological effects, including anti-tumor, anti-oxidant, anti-inflammatory, anti-fungal, anti-bacterial, and immunomodulatory properties (Khushboo et al., 2010). In a recent research, Babchi was indicated as a potential inhibitor of the viral cysteine protease of the COVID-19 (Mandal et al., 2021).

**Jasmine**/*Jasminum sambac* (L.) Aiton/“Moli,” “茉莉”

The leaves and roots of jasmine, *Jasminum sambac* (L.) Aiton, are an important component of TCM with the effect of clearing heat and removing excessive water. Jasmine has strong historical and cultural implications in China. Traditionally, flowers of jasmine can be ground into a powder that is used as a mask for detoxification, acne, and facial nourishment. Perfumes made of essential oils from jasmine are popular. Petals of jasmine can be used to make tea with a fragrant smell. Studies have indicated that jasmine exhibits activities of gastroprotective (Alrashdi et al., 2012), anti-inflammation, analgesia, and pyretic (Sengar et al., 2015). Presently, jasmine is not included in PPRC. In Iran, jasmine is widely grown as a valuable plant used for home ornament, landscaping, as well as for essential oils and medicine (Ghasemi Ghehsareh et al., 2015).

*Carpesium abrotanoides* L./“Tianmingjing,” “天名精”

*Carpesium abrotanoides* L. has been used as a TCM for a long time in China. It is mainly used for sore throat, tonsillitis, bronchitis, external treatment of traumatic bleeding, furunculosis and pyogenic infections, and insect and snake bites (Qian et al., 2019). It has been shown that *C. abrotanoides* mainly contains sesquiterpene lactone components, with anti-bacterial and anti-inflammatory, anti-viral, insecticidal, immunosuppressive, and other pharmacological effects (Liu et al., 2010; Pu and Pu, 2012). *C. abrotanoides* is not included in the PPRC.

**Saffron**/*Crocus sativus* L./“Fanhonghua,” “番红花”

Saffron is a famous spice in the Middle East and Europe. In China, saffron is the most representative medicine inherited from Persia to China along the Silk Road. The dried stigma of *Crocus sativus* L. is now included in PPRC. It has the efficacy of treating depression and tranquilizing the mind, activating blood and resolving stasis, and cooling blood and removing toxins. Saffron contains more than 150 compounds, the most important of which are water-soluble carotenoids including crocetin and crocin. With a limitation of extremely low yield and labor extensive handling, saffron production usually comes with high costs. However, it continuously attracts numerous studies for various health-related diseases in human subjects, such as inflammatory status of elderly hypertensive men (Mojtahedi et al., 2022), obese men with type 2 diabetes mellitus (Hooshmand Moghadam et al., 2022), chemotherapy-induced peripheral neuropathy (Bozorgi et al., 2021), and it has been shown to increase happiness as it reduces depression (Moghadam et al., 2021). In Iran, the dried, red stigmas of the *C. sativus* are thought to be a stimulant with anti-spasmodic action and are also a favorite coloring spice in food (Parsa, 1959b).

### 3.2 The category of vegetables

**Dill**/*Anethum graveolens* L./Shiluo, “莳萝”

Dill is mainly used in clinical treatment of digestive system diseases, such as stomach pain, indigestion, halitosis, and flatulence. It can also be used to treat child hiccups and promote lactation in lactating women (Tian, 2012). Essential oils made out of the dill contain volatile compounds such as carvone, limonene, and α-phellandrene. It also produces a great amount of flavonoids, phenolic compounds, cardiac glycosides, and terpenoids (Goodarzi et al., 2016), which have anti-bacterial, anti-oxidant, anti-gastric ulcer, and cholesterol-lowering effects (He and Huang, 2011). The essential oil of dill has good bacteriostatic activity and can be used as a powerful natural insecticide (Osanloo et al., 2017). Dill tinctures have the function of increasing aroma and enriching tobacco fragrance, and therefore, can be used in the tobacco industry (Zhan et al., 2007). Dill is a popular spice in Iran for condiment and carminative and is sold in the bazaars. When distilled, dill essential oil and dill water can be made at the same time for medicinal uses and drinking. In Iran, the leaves of dill can be a condiment to cook with rice to restore lost appetite (Parsa, 1959a). The Anethum tablet in Iran is a market medicine for hypolipidemic treatment, which is composed of four herbs, *A. Graveolens* (68%), *Cichorium intybus* L. (5%), *Fumaria parviflora* Lam (5%), and *Citrus* × *aurantiifolia* (Christm.) Swingle (4%) (Oshaghi et al., 2016).

**Spinach**/*Spinacia oleracea* L./“Boleng,” “菠薐”

Spinach is a common vegetable rich in carotene, vitamin C, protein, calcium, iron, and other minerals. In TCM, it has the functions of heat clearing, dryness moistening, and invigorating of the blood circulation. Although spinach is widely used as a vegetable worldwide, some studies have shown its pharmacological actions as it may help with some diseases, such as metabolic syndrome (Panda et al., 2017), cartilage degeneration and subchondral bone deterioration (Kothari et al., 2020), and also the postmenopausal osteoporosis (Adhikary et al., 2017). In Iran, spinach is mainly cultivated and consumed as a vegetable like in other countries; however, the fruits of spinach have demulcent and diuretic functions, and are sometimes prepared in order to reduce the bowel inflammation (Pasrsa, 1960).

### 3.3 The category of fruits

**Phoenix date**/*Phoenix dactylifera* L./“Wulouzi,” “无漏子”

Phoenix date is the fruit of the *Phoenix dactylifera* L, which grows in the high temperature and arid desert areas, such as Iraq and Iran. It tonifies the interior and reinforces the qi, eliminates phlegm, and stops coughing. Phoenix date has a high nutritional and medicinal value, and it is rich in vitamin A and vitamins B1 and B2. It has the function of protecting visual acuity, strengthening nerves, and softening blood vessels. In addition, other studies have shown that date also has a certain sedative effect, which has a certain effect on psychological panic and fear, as well as neurological disorders caused by hyperthyroidism (Zhou, 1986). The dates are also shown to relieve the kidney calculi (Mohammadparast Tabas et al., 2021), hypolipidemic and atherosclerosis (Bouhlali et al., 2020), or labor pain during delivery (Bagherzadeh Karimi et al., 2020).

Phoenix dates are widely planted in Iran. It is now cultivated in tropical areas of Fujian, Guangxi, Hainan, Guangdong provinces, and other places in China. The dates are usually used as food in Iran and China. They are not included in the PPRC. In Ahvaz city of Khuzestan province of Iran, a drink made out of phoenix dates is used for colic and sunstroke (Pasrsa, 1960).

**Fig**/*Ficus carica* L./“Wuhuaguo,” “无花果”

The medicinal part of fig is the fruit of *Ficus carica* L. In addition, the roots and leaves can also be used as medicine. The CMM describes the efficacy of the fruits as moistening lungs to stop coughing. Its roots and leaves are good for enteritis and diarrhea. Currently, figs are mainly consumed as fruit and are not included in the PPRC. Figs are rich in amino acids, vitamins, proteins, inorganic salts, and other beneficial ingredients. The fig is good in relieving hyperglycemia and hyperlipidemia (Ramadan et al., 2021), anti-stress of the skin (Dini et al., 2021), ulcerative colitis (Zou et al., 2020), anti-inflammation, and anti-proliferation (Liu et al., 2019). The fig fruit was used as medicine for anti-hemorrhoids, laxation, and tonic in Iran (Buso et al., 2020).

Golden shower/*Cassia fistula* L./“Alebo,” “阿勒勃”

Golden shower is a tree that grows up to 9–12 m high with 3–5 m wide upon maturity. Based on the TCM theory, *C. fistula* reduces invading pathogenic heat and wind resting in the heart and diaphragm. In southern China, *C. fistula* is mostly used in gardens and parks for ornamental purposes. *C. fistula* is not included in PPRC, however, it is recorded in Tibetan and the Dai nationality medical books in China. In Tibetan medicine, it is used for clearing heat, treating liver disease, and constipation, and reducing swelling of the limbs. The villagers of Dai nationality use it for anti-bacterial effects and for relieving constipation (Xie 1986). Modern pharmacological studies have disclosed that *C. fistula* is effective against *Leishmania donovani* (Tabrez et al., 2021) and type 1 diabetes (Indu et al., 2021). It has anti-oxidant actions and prevents neurodegeneration (Thabit et al., 2018). The chemicals in *C. fistula* are mainly flavonoids, anthraquinones, and disaccharides (Tan et al., 2018).

**Pistachio**/*Pistacia vera* L./“Ayuehunzi,” “阿月浑子”

Pistachios are one of the four major nut species in the world. Its dried fruits are rich in fat and various nutrients. Pistachio can moisturize intestines to loosen stools and help body detoxification. The nuts are also known to be a highly nutritious medicine and can treat microbial infection (Ozcelik et al., 2005), inflammation (Paterniti et al., 2017), oxidation (Shi 2014), anemia (Bailey and Stein, 2020), malnutrition (Bailey and Stein, 2020), chronic diarrhea (Sarkhail et al., 2020), wounds (Sarkhail et al., 2020), and obesity (Xia et al., 2020). Pistachios are currently consumed as food in China and are not included in the PPRC or other local pharmacopoeias. In Iran, *Pistacia* spp. contains a few species and all their fruits are consumed as food. The fruits of *P. vera* are considered as a tonic in Iran and the outer husk of the fruit is used to treat dysentery (Pasrsa, 1960).

**Chinese olive**/*Canarium album* (Lour.) DC./“Ganlan”, “橄榄”

Chinese olive is different from the European olive or common olive, *Olea europaea* L, which is a common tree in the Mediterranean Basin for olive oil or food. The dried ripe fruit of *Canarium album* (Lour.) DC. was included in PPRC. In TCM, olive helps produce body fluid, relieves restlessness, and quenches thirst and it is good for relieving throat pain. Chewing the fruit and swallowing the juice also helps to detoxify toxins from all kinds of fishes and turtles. Studies have proven that the chemicals of the fruits exert efficacy against some diseases, such as benzofuran neolignans for anti-inflammation (Li et al., 2022b), methyl brevifolincarboxylate (Chen et al., 2020b) and a polyphenolic compound isocorilagin (Chen et al., 2020c) both for influenza. The ethyl acetate fraction of Chinese olive was shown to regulate metabolic dysfunction in diabetes (Yeh et al., 2017).

**Jackfruit**/*Artocarpus heterophyllus* Lam./“Boluomi,” “波罗蜜”

Jackfruit is a famous tropical fruit with delicious flesh and a pleasant aroma. It is rich in sugar, proteins, vitamins, minerals, carbohydrates, and fatty oils (Gupta et al., 2022). In China, jackfruit is mainly consumed as fruit and is not included in the PPRC. In TCM theory, jackfruit is said to act as a tonic and reinforces the qi. It makes people feel happy and vigorous and can alleviate hunger. Recently, studies have discovered many active components with various pharmacological benefits from the jackfruit. Polysaccharides from jackfruit modulated the gut microbiota and they improved the production of the beneficial short-chain fatty acids (Zhu et al., 2021). Coumarin analogs from the jackfruit showed potential activities against inflammation and HIV (Tao et al., 2021). A fraction mostly containing artocarpin produced from jackfruit was effective in inhibiting the colorectal cancer cells (Morrison et al., 2021). Oxyresveratrol from jackfruit was shown to inhibit the production of melanin that is directly related to pigmented age spots and blemishes (Li et al., 2020).

**Grape**/*Vitis vinifera* L./“Putao,” “葡萄”

Grapes are now a fruit widely cultivated all over the world with high nutritional value. Grapes are also used as raw materials for making wine (not included in PPRC). In TCM theory, it has the effect of supplementing qi and blood, benefiting the kidney and liver, generating liquid, strengthening muscles and bones, stopping cough and eliminating vexation, and promoting urination. In addition to being used as a fruit, there are huge varieties of grapes for wine and beverage. The rich phytochemical composition of grape makes it an attractive object for pharmacological exploration. Through a systematic review, grape seed extract, which contains resveratrol and other anti-oxidant chemicals, may be beneficial for people with cardiovascular diseases (Foshati et al., 2022). The seed extract was effective to prevent cancer metastasis (You et al., 2021). The proanthocyanidin-rich fraction of the grape seeds attenuated memory impairment in rats (Osuntokun et al., 2021). The grape leaf extract exhibited strong anti-inflammatory and anti-vascular leakage during vein oxidative damage (Seo et al., 2021). The leaf extract can also reduce obesity in a mice model (Meng et al., 2021). Ampelopsin A, a major compound of grape, was found to ameliorate the cognitive and memory capability in neurodegenerative diseases (Hong et al., 2021). In Iran, although mostly used for fruits, dried grapes were also served as medicine with properties of demulcent, laxative, and cooling functions (Pasrsa, 1960).

### 3.4 The category of woods

**Agarwood**/*Aquilaria malaccensis* Lam./“Mixiang,” “蜜香”

Agarwood is regarded as being precious as it is favored by many fragrance enthusiasts because of its special fragrance. Shizhen Li in the CMM concluded that Mixiang is one species of Chenxiang (Chinese Eaglewood). It has similar therapeutic functions to Chenxiang. The name of Chenxiang is derived from its characteristics of sinking in water. When the resin content in Chinese eaglewood is higher than 25%, regardless of its form (tablet, block, or powder), it will sink into water. The agarwood mainly contains volatile chemicals such as sesquiterpenes, 2-(2-phenylethyl) chromone, aromatic, and flavonoids. Although it is believed that the agarwood has analgesic, anti-diarrhea, neuroprotective, anti-inflammatory, and anti-bacterial activities (Huo et al., 2018), thorough studies still fall short.

**Marking nut tree**/*Semecarpus anacardium* L. f [Anacardiaceae]/“Poluode,” “婆罗得”

The marking nut tree looks like a Chinese willow tree, and the seeds look like castor beans (*Ricinus communis* L*.*). It is also an important Ayurvedic medicine. In TCM theory, it warms the interior, tonifies the waist and kidney. It can dye hair and beard in black. It is not included in PPRC. Pharmacological studies have disclosed the medicinal values of the marking nut tree. The stem barks, or the nuts of the tree have been shown to possess potential against diabetes and inflammation in a rat study (Khan et al., 2013; Ali et al., 2015). The nut milk extract demonstrated remarkable hypolipidemic activity in hypercholesterolemic rats (Vinayagam et al., 2012). The methanol extract of the stem bark promotes wound healing and this was shown using a rat model (Lingaraju et al., 2012). The pericarp can be prepared like tea to relieve flatulence following severe piles in Iran (Pasrsa, 1960).

**Myrrh**/*Commiphora myrrha* (T.Nees) Engl./“Moyao,”“没药”

Myrrh is the dry resin from *Commiphora myrrha* (T.Nees) Engl. As an important TCM with a long history, however, myrrh is not produced in China. According to our research, myrrh was used as a component in 75 formulas out of 1605 TCM formulas registered in the current PPRC, proving its importance in TCM (data not published). In TCM theory, myrrh removes blood stasis, eliminates swelling, relieves pain, and promotes muscle growth. It falls within two categories in PPRC: natural myrrh and colloidal myrrh. Natural myrrh is of irregular granular clump with different sizes, the larger diameter can up to 6 cm or more. Natural myrrh has a special aroma with a bitter taste and slightly pungent smell. The colloidal myrrh is made of irregular lumps and granules with a brown surface, which stick into lumps of different sizes. Colloidal myrrh is opaque, solid or loose in texture with a specific aroma. The main chemical constituents of myrrh are monoterpene, ploidy, triterpenoids, steroids, and lignin (Ge and Zhang, 2019).

Studies have disclosed the modern pharmacology of myrrh. Water extracts and polysaccharides of *C. myrrha* are effective to treat the osteoporosis in postmenopausal women (Hwang et al., 2021). A 10 min sitz-bath of myrrh extract greatly relieved the wound healing of the episiotomy in a clinical trial (Faraji et al., 2021). *C. myrrha* can treat the diabetes mellitus and the resin solution stimulates insulin secretion (AlRomaiyan et al., 2021), and anti-microbial activity (AlMadi et al., 2019), human heterophyiasis with the gastro-intestinal troubles with parasitosis (Massoud et al., 2007). In Iran, myrrh is used as a stomachic and for lumbago (Parsa, 1959b).

**Benzoin**/*Styrax tonkinensis* (Pierre) Craib ex Hartwich/“Anxixiang,” “安息香”

Benzoin or benjamin is the dried resin of *Styrax tonkinensis* (Pierre) Craib ex Hartwich, a semi-deciduous tropical tree that can grow up to 20 m high. The outgoing resin is collected and dried when the tree stem is naturally damaged or cut apart in the summer or autumn. Benzoin is included in the PPRC. It is divided into two types: Thai benzoin and Sumatra benzoin. The resin content of benzoin is as high as 70–80%, and the main component of the resin is volatile vanillic acid. In addition, lignins, terpenoids, and steroids were obtained from the studies on the chemical compositions of the genus *Bemberine* (Zhang et al., 2014). Studies on pharmacological activities showed that benzoin mainly has anti-matrix metalloprotease-1, anti-ulcer, antioxidant, anti-complement, anti-bacterial, and other activities (Lei et al., 2012).

**Ferula**/*Ferula sinkiangensis* K.M. Shen or *Ferula fukanensis* K.M. Shen/“Awei,” “阿魏”

The PPRC stipulates that the medicinal material of this product is the resin of *Ferula sinkiangensis* K.M. Shen or *Ferula fukanensis* K.M. Shen. Both plants are native to Xinjiang, China and they are not present in Iran. Most likely the species in old times was classified as *Ferula asafoetida* (Falc.) H. Karst, which is native to Iran. In Iran, Ferula is used to treat spasmodism, helminth, carmination, and constipation (Iranshahy and Iranshahi, 2011). In TCM theory, Ferula has the effects of regulating qi and eliminating swelling, fatigue, and phlegm. Ferula promotes blood circulation and excites nerves. A pharmacological study in India showed the safety and efficacy of Ferula on functional dyspepsia (Mala et al., 2018). In China, studies on *F. sinkiangensis* and *F*. *fukanensis* provide more pharmacological advances. Kellerin is specific compound from *F. sinkiangensis* that was verified to improve the neuroprotective effects in a rat model (Mi et al., 2021). Fractions of *F. sinkiangensis* exerted anti-oxidant and anti-tumor effects in a cell study (Zhang et al., 2015). Sesquiterpenoids from *F. fukanensis* inhibited nitric oxide production (Motai and Kitanaka, 2005).

**Aloe**/*Aloe vera* (L.) Burm. f. or *Aloe ferox* Mill./“Luhui,” “卢会”

Aloe, originally growing in hot, dry climates, is a popular, succulent plant that is of horticultural importance. Aloe in the PPRC is a dried product of concentrated juice from *Aloe vera* (L.) Burm. f., *Aloe ferox* Mill., or other close species. Aloe is used to treat skin diseases, constipation, indigestion, and worms in TCM. The thick juice from *A. barbadensis* leaves is a good moisturizer, especially in cosmetics. A study has disclosed that a compound, aloin A, might be the bioactive agent that prevents infection of skin wounds when aloe is applied as a topical treatment (Donkor et al., 2020). The dry aerial parts of *A. barbadensis* can significantly restore the integrity of the hepatocytes toxified by carbon tetrachloride (Chandan et al., 2007). Components from *A. barbadensis* restore the metabolic and reproductive comorbidities of polycystic ovary syndrome (Dey et al., 2022). *A. ferox* is also called Cape aloe or bitter alone in South Africa, which is rich in chemicals of flavones, anthraquinones, anthrone-glycosides, and phenolic compounds (Parsa, 1959a). The laxative effect, skin and wound healing, anti-oxidant, anti-inflammation, anti-microbes, anti-cancer, anti-malaria, and permeation-enhancing function, as well as some adverse effects including vomiting were elaborately reviewed (Chen et al., 2012).

In Iranian medicine, aloe is applied as an anti-fever, anti-infection, and wound healing reagent (Dey et al., 2017). Nowadays, this medicinal plant is frequently used in the field of cosmetology (Haghani et al., 2022).

**Nutgalls/** *Quercus infectoria* G. Olivier/“Wushizi,” “无食子”

Nutgalls are the cecidum of a special larva (*Cynips tinctoria* Olivier) parasitized on the young branches of *Quercus infectoria* G. Olivier. Unpierced cecidum is mainly used for medicinal purposes. Nutgalls are rich in gallotannin (50–70%), followed by gallic acid (2–4%), propionic acid, and resin. It has the effect of strengthening teeth and fixing gums, clearing blood and relieving pain, and inhibiting bacteria. It is a common medicine used in Uyghur as an enema in the treatment of chronic ulcerative colitis (Huang and Fan, 2003). However, it is currently not included in the PPRC.

**Frankincense**/*Boswellia sacra* Flück/“Xunluxiang,” “薰陆香”

Frankincense is a resin that is obtained from bark exudates of *Boswellia sacra* Flück. Its shape and aroma are similar to turpentine. In TCM, frankincense is used for chest, heart, stomach, and other body pains, and it is also used for wounds, carbuncles, and skin ulcers. It has been demonstrated that *Boswellia sacra* Flück contains a wide range of chemicals that are components of its volatile oils, mainly diterpenoids and triterpenoids. These chemicals have pharmacological properties of anti-inflammation, anti-diabetes, and anti-cancer and are useful for cardiovascular and neurodegenerative diseases (Huang et al., 2022). Frankincense is included in the PPRC. *Boswellia sacra* Flück is not grown in China, so these products must be imported from other countries but the species is not grown in Iran where it is still being used as a medicine as well.

**Borneol**/*Cinnamomum camphora* (L.) J. Presl/“Longnaoxiang,” “龙脑香”

Borneol is the product of crystals obtained by steam distillation of the shoots and leaves of *Cinnamomum camphora* (L.) J. Presl. Borneol is a common Chinese medicinal material, and there are four kinds of borneol contained in the National Drug Standards of China (Li et al., 2013). Among them, natural borneolum and synthetic borneolum are included in the PPRC. According to our analysis, a total of 168 TCM formulas registered in the PPRC use borneolum as a component. The borneolum is dispensable in the TCM formula for anti-inflammation, anti-microbes and it is included in many TCM formulas for increasing the permeability of other components. The seed kernel extract and the essential oil of *C. camphora* were demonstrated to hold anti-inflammatory potential (Xiao et al., 2021; Zhang et al., 2021). The seed kernel oil of *C. camphora* improved lipid metabolism by reducing the body fat mass of obese rats (Fu et al., 2016). The borneol, as a pure compound, can be used to enhance the radiosensitivity of glioma tumor (Li et al., 2021), for neuroprotection in cerebral ischemia (Xie et al., 2022), and for the cardio-cerebrovascular diseases (Liu et al., 2021).

The application of borneol in TCM can be dated back a thousand years ago. All the natural borenolum was imported from overseas until the wildly grown tree of *C. camphora* was discovered in 1990s in south China. Currently, *C. camphora* is a medicinal tree in Iran.

**Black Myrobalan, Chebule**/*Terminalia chebula* Retz./“Helile,” “诃黎勒”

Chebule is the dry ripe fruit of *Terminalia chebula* Retz., and now it is included in the PPRC. *T. chebula* is distributed in Iran. Chebule is used for chronic diarrhea and dysentery, bloody stool and rectal prolapse, lung deficiency, panting and coughing, chronic cough, angina, and hoarseness. Modern pharmacological studies have confirmed that its main chemical components include tannins, phenolic acids, triterpenes, and flavonoids. These components have many effects, such as anti-oxidant, neuroprotective, anti-tumor, anti-viral, and anti-bacterial activity. A combination formula containing *Commiphora mukul* Engl., *Commiphora myrrha* (T.Nees) Engl., and *T. chebula* was comparable to metformin in treating diabetic rats, suggesting its potential remedy in diabetes (Sotoudeh et al., 2019). The fruit of *T. chebul*a, chebule, is frequently used for skin hyperpigmentation in Iran (Khazaeli et al., 2009). The young, unripe nuts which turn black on drying, called Halilah-i-siah or black myrobalans, are mainly used in medicine as a strong purgative for treating stomach pains (Pasrsa, 1960).

**Large fruited elm**/*Ulmus macrocarpa* Hance/“Wuyi,” “芜荑”

The product is made by fermenting and drying the fruit pods of *Ulmus macrocarpa* Hance, which are added with auxiliary materials such as elm bark flour. The CMM recorded that it disperses invading pathogenic factors resting with five Viscera. It also disperses toxins of febrile diseases circulating in the skin and joints. It kills worms and helps digestion. The large-fruited elm is not included in the PPRC.

*U. macrocarpa* is widely distributed in Korea and north China. However, only the same genus plant but not the same species is present in Iran. Studies have shown that extracts of *U. macrocarpa* increase the host immunity by modulating the gut microbiota in human subjects (Kim et al., 2021). A taxifolin flavonoid glycoside extracted from *U. macrocarpa* ameliorates the osteopenic disease (Wang et al., 2022). The root bark of *U. macrocarpa* exerts anti-hypertensive, vasorelaxant, and anti-oxidant properties with chronic treatments in rats. (Oh et al., 2008).

**Ebony**/*Diospyros ebenum* J. Koenig ex Retz./“Wumu,” “乌木”

Ebony is a slow-growing and evergreen tree with a potential to grow as high as 30 m. It is distributed in south China and Iran. In TCM, the product is the wood of *D. ebenum*. It is used for detoxifying toxins and treating cholera with vomiting and diarrhea (Perera et al., 2018). In addition to being used as medicine, it is also a very precious wood with high collection value. It is not included in the PPRC. Little modern pharmacology on ebony is conducted.

### 3.5 The category of metals, stones, and minerals

**Silver ore**/“Xilinzhi,” “锡吝脂”

Xilinzhi, also known as Persian silver, is the silver ore produced in Persia. In old times, it was mainly used to treat pterygium and it is currently not included in the PPRC. No report of using silver ore in the current TCM.

**Atacamite**/“Lvyan,” “绿盐”

Atacamite is the copper ore and the main component is copper chloride. It is often mixed with aluminum, iron, calcium, and magnesium. The crystal is columnar or plate-shaped with vertical stripes. In old times, it was used for treating inflamed eyes with shedding of tears, and dim vision with profuse secretion and slight pterygium. Atacamite is highly toxic, and when mistakenly swallowed it will stimulate gastric mucosa that causes vomiting and abdominal pain. It is not included in the PPRC and no report has been found on its usage in the current TCM.

**Alum stone**/“Fanshi,” “矾石”

There are five kinds of alum stones listed in the CMM and these are differentiated by the color: Baifan (white, Alumen), Huangfan (yellow, Fibroferritum), Lvfan (green, Melanteritum), Heifan (black alum), and Jiangfan (red, Alumen perparata). Presently, the PPRC contains Alumen and Melanteritum. In TCM, the alumen treats itching and worms for topical treatment and it stops bleeding by oral administration.

**Sulfur**/“Shiliuhuang,” “石硫磺”

The massive sulfur is a light yellow crystal that has an unpleasant stench. In TCM, sulfur for external use can kill worms to stop itching, treat scabies, eczema, and skin pruritus. For internal use, it is indicated to nourish fire and invigorate Yang, in addition to strengthening sinews and bones, and to relieving constipation. Sulfur benefits qi and stops bleeding, dispels phlegm and reduces panting. However, sulfur is toxic so it is suggested not to be taken in large quantities or for a long period. Sulfur is not contained in the PPRC. Limited cases of sulfur are used in the TCM formula. Sulfur is a very efficient fumigation reagent and is widely adopted in the TCM material treatment. However, the excessive residue of sulfur is forbidden and it is a monitored substance in the quality control of TCM material.

**Salt**/“Guangmingyan,” “光明盐”

Salt is a natural crystal that mainly contains sodium chloride. In TCM, it is primarily used for dispelling pathogenic wind for improving eyesight, digestion and accumulation, and detoxification. It is now only a food additive and not a medicine in China. It is not often to be used in TCM formulas but is used in some applications of moxibustion or massage. Although currently sodium chloride is commonly used as an essential condiment for the maintenance of concentration and osmotic pressure crossing cell membrane, treatment with additional sodium chloride is necessary for diseases characterized by lack of salt including cystic fibrosis (Keivanfar et al., 2020), congenital chloride diarrhea (Wedenoja et al., 2008), and small bowel ostomies (Trautmann et al., 2020). No clue is found to connect the traditional and current remedies with the salt.

**Litharge**/“Mituoseng,” “密陀僧”

Litharge is a red oxide mineral of lead, belonging to the tetrahedral system. Litharge mainly contains lead oxide, which has certain toxicity. Internal use causes lead poisoning. In TCM, litharge treats regurgitation, diabetes, malaria, and dysentery. It also stops bleeding, kills parasites, and removes food retention. It is good for treating sores, eliminating swelling and attacks of noxious agents, dispersing bromhidrosis, and dyeing the hair black. Litharge was externally used to treat eczema, sore poison, burns, scalds, injuries from falls and other surgical diseases. Internal use includes treating diarrhea, malaria, epilepsy, and other diseases. Litharge is currently not included in the PPRC. No report of using it in the current TCM.

**Iron**/*Ferrum*/“Tie”, “铁”

Iron is a grayish-black metal, mainly produced from hematite, limonite, and magnetite. The CMM claimed that iron could dissipate extravasated blood and eliminate erysipelas. Iron is used as the main medicinal treatment for incised (metal-inflicted) wounds, chest and diaphragm pain due to being blocked by gas, and retention of food. However, the CMM also stated that it could damage the lung and injure the liver and kidney. Iron is not included in the current PPRC, but in the previous version of PPRC; red, yellow, brown, purple, and black ferric oxides were included. No reports of any use are included in the current TCM.

**Gold**/“Jin,” “金”

Gold was recorded in many Chinese ancient books as a medicine. However, the gold needs to be made into gold foil before it can be used as medicine. The internal use of gold foil has the effect of leading to tranquility and protecting the liver. The external use is said to detoxify and treat furuncle. Clinically, it is mostly used for treating convulsive epilepsy and insane and disquieted heart spirit. Gold is not included in the PPRC. No report of its current use is now indicated in TCM.

**Silver**/“Yin,” “银”

Silver has been a medicine in China for thousands of years. The needles for acupuncture and moxibustion were made of silver. In terms of medicinal materials, silver flakes were mainly used for epilepsy and mania, palpitation and trance, treating pox sores, and sudden loss of consciousness in children. Nowadays, silver is mostly used for anti-infection, anti-bacterial, and anti-inflammation purposes. Sliver is not included in the PPRC.

**Copper**/“Chitong,” “赤铜”

In the CMM, it is noted that the debris of red copper falling off during forging was used for medicinal purposes, and it has the effect of connecting bones and tendons, dispersing blood stasis and relieving pain. Internal treatment can cure blood stasis obstructing the collaterals, and external treatment is implicated in curing bleeding wounds and for eyelid ulcerations. Copper has also been used as a dye to make hair black and remove odors. However, internal use is not necessarily advised as this can lead to stomach and kidney damage and so, copper is suggested not to be taken in large quantities or for a long time. Natural copper (pyrite, mainly containing iron disulfide) is recorded in the PPRC for treating injuries that may be associated with falls, fractures, contusions and strains, and pain due to stasis swelling.

**Lead**/“Qian,” “铅”

In TCM, lead is good for eliminating scrofula, carbuncles, and swelling. It improves eyesight, stabilizes the teeth, and blackens the hair. It is effective for female suffering absence or atresia of the vagina (a birth defect). It kills parasites and eliminates phlegm. It is also good for treating dysphagia, diabetes, and acute infantile convulsions. Consequently, drugs containing lead should be used carefully in clinical practice to ensure the safety. It is not included in the PPRC. Lead is a poisonous element and a high level of lead in the blood affects brain function and development. Currently lead is monitored in TCM material due to its poisonous nature.

**Coral**/*Corallium*/“Shanhu,” “珊瑚”

In TCM theory, coral has a similar medicinal efficacy to that of gold. Due to the specificity of the marine environment, corals have a variety of secondary metabolites and a wide range of biological activities. There are thus opportunities for the development of new pharmaceutical coral–derived ingredients in marine natural drug research. It is not included in the PPRC. At present, it is not common to use coral in TCM treatments.

**Cinnabar**/“Dansha,” “丹砂”

Cinnabar is a mercuric sulfide mineral, which played a major role in old Chinese alchemy to increase longevity. In TCM it clears the heart, calms the mind, brightens the eyes, and detoxifies the body. When used externally, it can inhibit skin fungi. Mercury is a heavy metal element that is highly toxic. Its presence in common TCM material is thus rigorously monitored and concentrations have to be exceedingly low and limited to ensure quality control aspects linked to safety and toxicity. Cinnabar is included in the PPRC; however, no formula is registered with it.

**Calamine**/“Luganshi,” “炉甘石”

According to the CCM, calamine is produced in Persia and is similar to gold in appearance. Calamine is a carbonate mineral calcite rhombozite, mainly containing zinc carbonate. It has a sweet and neutral taste and is non-toxic. Calamine is frequently used in ophthalmology and surgery. It has the effect of detoxifying, improving eyesight and removing nebula, dispelling excessive water, relieving itching, and healing sores. It is included in the PPRC. Many modern proprietary medicines contain calamine components, such as medicament for the eyes (Babao, Guangming). Calamine is not suitable for internal use and is frequently made into liniment or lotion with other drugs.

**Fibroferrite**/“Huangfan,” “黄矾”

The CMM mentioned that fibroferrite ore produced in Persia has a golden thread when cut open. Fibroferrite was used as medicine before the Song Dynasty but was rarely used after that, and it is not included in the PPRC.

### 3.6 The category of fowls

**Ostrich**/*Struthio camelus*/“Tuoniao,” “鸵鸟”

Ostrich originally lived in the semi-arid desert areas of Africa. According to historical records, in 101 AD, the Sabbath Dynasty presented lions, ostriches, and other animals as gifts to Chinese emperors. It is not included in the PPRC. It is a zoo animal in China and there is no report of using it in the current TCM.

## 4 Discussion

The earliest footprints of Persian businessmen appeared in the Chinese cities of Changan, Luoyang, Guangzhou, and Quanzhou. The book, *Taiping Guangji* (first edition in 978 of Song Dynasty), recorded a large number of Persian merchants who settled down in Chang’an to trade the jewelry and spice. At the same time, China’s ceramics, paper, silk, tea, porcelain, sericulture, and other expertise were also introduced to Persia, which greatly impact the empire. Yunsheng Hu (Hu and Zhang, 1996) in the study of foreign drugs in the Tang Dynasty indicated that the aromatic drugs are a bulk trade commodity, and a large number of them come from Persia and the Seljuks. The import and export of Persian drugs mainly rely on merchants all over the country. In addition to importing its own drugs, the Persians also collect drugs from foreign countries to trade in China.

Foreign medicines enrich the varieties of traditional Chinese medicines and meet people’s needs to treat diseases and maintain health. There are a few fates of these medicines in China. 1) Some foreign drug resources spread to China, over time, they have gradually adapted to the cultivation environment in China and have been included in the “Chinese herbal medicines,” becoming China’s localized medicine. Saffron, myrrh, and olibanum are as such medicines that are all included in the PPRC for treating diseases even though these medicines are still largely dependent on imports from Iran or other countries. 2) Some medicinal materials are no longer included as TCM materials, but are treated as common food through long-term of experiencing, recognition, and understanding. As mentioned earlier, dill and spinach became common vegetables not only in China but also in the rest of the world. Fig, grape, jackfruit, phoenix date, and pistachio are popular fruits with good taste and nutrition. Jasmine, olives, and other fragrant medicines are primarily being used as raw materials for processing into many products including perfume, essential oil, and skin care products, which can not only make full use of the value of these plants, but also meet the diverse needs of people. Based on the theory of TCM, the aforementioned fruit and vegetables can be classified in a group of food and also medicine due to their tonic factors. 3) In addition, some medicinal materials, due to excessive toxicity, are listed outside the range of commonly used medicinal materials. This part of the medicinal materials is mostly concentrated in the category of metals, stones, and minerals. For example, lead and litharge are no longer used as medicinal materials. Cinnabar, copper, and calamine are also extremely restricted in clinical use. Lead and mercury are closely monitored in the standardization of quality control of all TCM materials for contamination of heavy metals.

Of interest, 25 of the 38 TCM medicinal plants now are cultivated or wild growing in China but they are not found in Iran. There are some possible explanations for this. First, the authentification of material used in TCM is changing with time. In old days without scientific names, it is possible that different plant species could easily be confused, especially if they share similar medicinal efficacy. For example, natural indigo, the three plants, namely, *S. cusia*, *P. tinctoria*, and *I. tinctoria* subsp*. tinctoria* show similar treatments and people treat them the same but it might be *I. tinctoria* subsp*. tinctoria* which was the only one introduced from Persia to China. Second, some of the medicinal plants from Persia might have been replaced by the ones that were naturally found in China. When plants have close pharmacological effects, harvesting from nearby areas is more economical than obtaining plant materials from faraway places that are ten thousand of kilometers away. If this is the case, some of species that are naturally distributed in Iran might be valuable for drug discovery as they have found use in China, such as close species of *Cullen corylifolia* (L.) Medik., and *Terminalia chebula* Retz., are all found in Iran. Another example is of the Ferula species. *F. sinkiangensis* or *F. fukanensis* are the two species listed in the PPRC but these two plants were discovered in the 1950s in China and now they are endangered with limited production. For a long time, the Ferula imported from Persia must be the close species *Ferula assa-foetida* L. or other species in Iran. Re-connection of the China–Iran bilateral authentification of the medicine may help to utilize the rich resources of Ferula in Iran. Third, some of the medicinal plants are not distributed in Iran or even in old Persia, but they were introduced by the Persia businessmen and brought to China. It is confirmed that Iran does not produce myrrh and for a long time and the great demand of the Chinese market might be provided by Persian commercial men, who obtained it from other places. It was until recent decades that China started importing the myrrh from Ethiopia and nearby countries. In this way, the Silk Road in the old times may have extended to Africa by Persia (Figure 1). We are now collecting more evidence to verify the connection of East Asia to the central Africa of the Silk Road.

There are 24 TCMs, consisting of 38 medicinal plants and four stones, studied in this study and were included in the 2020 edition of the Pharmacopoeia of the People’s Republic of China. The pharmacopoeia is a drug code established by the state to ensure the quality and safety of the medicine. Drugs recorded in the pharmacopoeia are commonly used as medicinal materials and are permitted in use in the clinical practice. In Table 3, these drugs were counted to provide reference for the use of these medicinal materials. Since ancient times, the Chinese had been actively absorbing outstanding foreign culture. The development of communication between China and abroad spreads Chinese civilization to the world and brings back the civilizations from all over the world. The CMM has played an important role in the TCM development. Until today, the inclusive thinking of Shizhen Li on foreign culture is something we should learn.

The introduction and cultivation of exotic drugs is an important part of the development history of Chinese herbal medicines. Persia was an important country on the Silk Road. Looking back to the date when Shizhen Li was writing the book and even earlier, the introduction of traditional medicine benefited both sides. With saffron as an example, even though it is now sporadically cultivated in China, much more is imported to China from Iran, perhaps continuously lasting over two thousand years. It is not because it cannot grow in China. It is because in the Chinese medicinal culture Persia produces the best quality saffron even though it is treated as a native medicine. The peaceful development of China and Persia has a positive impact on the politics, economy, and culture of the two territories. It is the best example and most valuable legacy of the Silk Road on the sharing of traditional medicinal resources. It shall inspire us to peacefully develop a collaborative relationship to share the advancement of the human civilization.

## Footnotes

1 Here the common name, scientific name, Chinese phonics, and characters for each medicine are shown. The Chinese phonics and characters are from the CMM. The current name is shown in Table 1 and 3.
